# Chemical Composition and Bioactive Properties of Commercial and Non-Commercial Purple and White Açaí Berries

**DOI:** 10.3390/foods9101481

**Published:** 2020-10-16

**Authors:** Fernanda V. Matta, Jia Xiong, Mary Ann Lila, Neil I. Ward, Mónica Felipe-Sotelo, Debora Esposito

**Affiliations:** 1 ICP-MS Facility, Department of Chemistry, University of Surrey, Guildford, Surrey GU2 7XH, UK; n.ward@surrey.ac.uk (N.I.W.); m.felipe-sotelo@surrey.ac.uk (M.F.-S.); 2 Plants for Human Health Institute, Animal Science Department, North Carolina State University, North Carolina Research Campus, Kannapolis, NC 28081, USA; jxiong5@ncsu.edu; 3 Plants for Human Health Institute, Food Bioprocessing and Nutrition Sciences Department, North Carolina State University, North Carolina Research Campus, Kannapolis, NC 28081, USA; mlila@ncsu.edu

**Keywords:** açaí, total polyphenol, total anthocyanin, antioxidant, elemental analysis, essential elements, biological activity, wound healing

## Abstract

Chemical composition analysis of açaí extracts revealed higher levels of total polyphenol content in purple açaí samples for both commercial (4.3–44.7 gallic acid equivalents mg/g) and non-commercial samples (30.2–42.0 mg/g) compared to white (8.2–11.9 mg/g) and oil samples (0.8–4.6 mg/g). The major anthocyanin compounds found in purple açaí samples were cyanidin-3-glucoside and cyanidin-3-rutinoside with total concentrations in the range of 3.6–14.3 cyanidin-3-glucoside equivalents mg/g. The oligomeric proanthocyanidins were quantified in the range of 1.5–6.1 procyanidin B1 equivalents mg/g. Moreover, açaí presented significant levels of calcium, magnesium, manganese, iron, zinc and copper, essential minor and trace elements, in comparison with other berries. All of the açaí extracts at 50 μg/mL potently inhibited the release of reactive oxygen species in lipopolysaccharide-stimulated RAW 264.7 murine macrophage cells, but none inhibited the release of nitric oxide. Furthermore, all the açaí samples demonstrated potential as wound healing agents due to the high levels of migration activity in human fibroblast cells.

## 1. Introduction

The açaí palm (*Euterpe* genus) is a native tree from the Amazon region, northern South America. There are 28 known species, but only two are commercially available, namely *E. oleracea* and *E. precatoria* [1]. The former, native from the Amazonas river estuary, is the most commercially valuable due to its supposed higher quality fruit compared to the latter from the Amazonas river basin [2]. The palm tree can grow up to 25 m in height [3]. Its fruit is a small black-purple berry, of which the seed accounts for 80 to 95% of the fruit and the edible purple pulp is only 1 to 2 mm in thickness [4]. There is also a rare different variety of açaí fruits that maintain the greenish colour after ripening, called white açaí [1]. White açaí is commercially available, but it is still mainly collected and consumed by the native people from the Amazon [5]. Although the white açaí has potential antioxidant capacity, there is a lack of research in the composition or possible health effects of these berries, in contrast to the purple variety [6,7].

The açaí is mostly consumed as a thick viscous juice, obtained by mechanical extraction of the pulp. For many years, it has been an important dietary source of nutrients for the Amazonian people, contributing up to 43% of their diet on a dry weight basis [8]. Brazil is the main producer and exporter of açaí, generating an estimated monetary movement of 9 billion dollars per year, exceeding the price per ton of soybeans and Brazil nuts [1]. Recently, açaí has gained popularity in South America as well as globally due to its classification as a “super fruit” [9]. The demand for açaí has been increasing in southern Brazil and it is usually consumed by the younger population due to its appeal as an energetic, healthy and nutritional product [4]. Unfortunately, the escalating demand for açaí and its relatively low processing yield have motivated frequent increase of product adulteration in the market place [10]. The present demand for açaí is mainly for the food industry, but it is expected that other activities, such as the development of nutraceuticals, cosmetics, and personal care products, will increase significantly in the near future.

Past reports on the chemical composition and bioactive potential of açaí vary widely, depending on harvest dates, geographical sites, incidence of adulteration, and the effects of processing [1,8,11]. Health-relevant features reported for açaí include anti-inflammatory [9], cardio protective [12,13], antioxidant [14] and anti-proliferative properties [15]. These biological properties have mainly been attributed to the high polyphenol content of the açaí berries [16]. It has been reported that the açaí berry is a good dietary source of potassium, calcium, magnesium and manganese K, Ca, Mg, Fe and Mn [17].

Only a few studies have been published concerning the potential of açaí to promote wound closure [11,18,19]. The process of human wound healing is very complex, but it can be divided into three main phases: inflammatory, proliferative and maturation [20]. The first occurs after body injury when many different inflammation processes can occur, such as pain and swelling. The proliferative phase occurs when the fibroblasts migrate from the tissue to the wound so as to close the injury. Then, finally at the last stage, collagen is deposited into the tissue [20]. There is no time frame for the duration of each one of the phases and the body can go back and forward in this healing process based on different factors. Positive factors that help to improve or accelerate the wound healing process are: vitamins A, C and E, iron, zinc and fats [21]. Several berries with high polyphenol content have shown a potential to improve the migration of human dermal fibroblasts [22,23], and commercial açaí berries could also demonstrate the same effect.

The aim of this work was to determine the chemical characterization and biological activities (antioxidant and wound healing) of different samples of açaí. In our previous research, we have investigated the effect of industrial process on polyphenols from açaí [11]. The unique feature of this research was further to evaluate the composition and bioactivities of non-commercial samples of both white and purple açaí berries in comparison with the commercially available açaí. Due to the worldwide increase in the consumption of açaí, this study was designed to advance the knowledge on the food composition and investigate the bioactivity of antioxidant nutrients of açaí pulp. This information is especially important for the population that heavily relies on açaí as part of their diet, and also for food industry that could develop numerous products based on the açaí composition and properties.

## 2. Materials and Methods

### 2.1. Plant Amaterial (E. oleracea)

Commercial samples were bought as pure açaí powder at local supermarkets in the United Kingdom (commercial UK) and Brazil (commercial SP). A frozen pulp commercialised as pure açaí was bought in São Paulo (Pulp SP) and freeze dried at Universidade de São Paulo. In addition, non-commercial purple and white whole açaí berries were obtained in natura directly from the Amazon region (Abaetetuba, Pará, Brazil, 1°43′46″ S and 48°52′27″ W) and were freeze dried at Universidade Federal do Pará. The production of açaí is an extractive activity from the floodplain of the Amazon forest, and there is no information available about the climate, growing or processing conditions of the samples. Therefore, the comparison between samples is not precise. The oil was extracted using the supercritical carbon dioxide method previously described [24] producing the de-fatted samples.

### 2.2. Extraction of Açaí Samples

Samples of 0.20 ± 0.01 g of ground freeze-dried açaí were extracted (in duplicate) with 5 mL of acidified 70% *v*/*v* High-Pressure Liquid Chromatography (HPLC) grade methanol in 0.5% *v*/*v* HPLC grade acetic acid (HAc, Fisher Scientific, Pittsburgh, PA, USA). The mixtures were homogenized using a Pro 250 homogenizer (Pro Scientific Inc., Oxford, CT, USA) for 10 min and centrifuged (Sorvall RC-6 plus, Asheville, NC, USA) for 10 min at 5000 rpm. The supernatant was transferred to a 25 mL volumetric flask. The resultant pellet of açaí was further extracted twice and the extracts were combined and diluted to a final volume of 25 mL. Each final solution was filtered using a 0.2 μm polytetrafluoroethylene (PTFE) syringe filter (Fisher Scientific, Pittsburgh, PA, USA) before analysis.

### 2.3. Chemical Analysis and Antioxidant Activity

#### 2.3.1. Chemicals

Folin–Ciocalteu reagent, sodium carbonate, gallic acid, aluminium chloride, quercetin, 4-dimethylaminocinnamaldehyde (DMAC), cyanidin-3-glucoside (C3G), and procyanidin B1 dimer were purchased from Sigma-Aldrich (St. Louis, MO, USA). All other chemicals and solvents were obtained from Fisher Scientific (Pittsburg, PA, USA).

#### 2.3.2. Total Polyphenol Content by Folin–Ciocalteu Assay

The total polyphenol content was determined using the Folin–Ciocalteu assay following the Singleton method [25] and adapted for a micro-plate assay. In a 96 well-plate, 75 μL of distilled water was added to each well (in triplicate) along with 25 μL of the diluted sample or standard, and 25 μL of diluted (1:1 *v*/*v*) Folin–Ciocalteu reagent. After 6 min, 100 μL of 7.5% sodium carbonate solution was added. The plate was then left in the dark for 90 min and read using a UV-Vis plate reader (SpectraMax^®^ M3, Sunnyvale, CA, USA) at 765 nm. The results are expressed in gallic acid equivalent (GAE), which was used as a standard reference.

#### 2.3.3. Total Flavonoid Content by AlCl_3_ Assay

The total flavonoid content was determined spectrophotometrically using the AlCl_3_ assay in a micro-plate assay [26]. In a 96-well plate, 100 μL of sample or standard was added to each well (in triplicate) in addition to 100 μL of 2% AlCl_3_ solution; the plate was mixed and left at 20 °C for 1 h. The absorbance was read using a UV-Vis plate reader at 415 nm. Results are expressed as quercetin equivalent.

#### 2.3.4. Total Anthocyanin Content by HPLC

The determination of the total anthocyanin content of the açaí extracts was performed using a high-performance liquid chromatography (Agilent 1200 HPLC, Santa Clara, CA, USA) instrument with a photodiode array detector (DAD) [27]. The separation was conducted using a reversed-phase Supelcosil-LC-18 column, with dimensions of 250 mm × 4.6 mm × 5 μm (Supelco, Bellefonte, PA, USA) held at a constant temperature of 30 °C with a flow rate of 1 mL/min. As a mobile phase, 5% formic acid in distilled water was used as solvent A and 100% methanol as solvent B. The step gradient used was 10%, 15%, 20%, 25%, 30%, 60%, 10%, and 10% of solvent B at 0, 5, 15, 20, 25, 45, 47, and 60 min at a flow rate of 1 mL/min. The quantification of the total anthocyanin content was based on the sum of the integrated anthocyanin peaks calculated against the standard curve of cyanidin-3-glucoside (C3G), presented as (C3G)/g dry weight.

#### 2.3.5. Total Proanthocyanidin Content (PAC) via DMAC Assay

The total proanthocyanidin (PAC) content was determined using the 4-dimethylaminocinnamaldehyde (DMAC) method adapted for a micro-plate assay as previously described [28], where the DMAC reacts with the terminal units of the PAC oligomers. In a 96 well-plate, 63 μL of diluted sample, standard or blank, was added to each well along with 189 μL of the DMAC reagent in triplicate. The plate was set on the plate reader at 640 nm to record the absorbance value of the wells, at one-minute time intervals for 30 min. The results are expressed as procyanidin B1 dimer equivalent that was also used as a standard reference.

#### 2.3.6. Antioxidant Activity by ABTS and DPPH Assay

Antioxidant activity of the açaí extracts was measured using the conversion of the 2,2′-azino-bis(3-ethylbenzothiazoline-6-sulphonic acid) or ABTS reagent to the radical cation ABTS+• by potassium persulfate that reacts with the antioxidant compounds present in the extracts, such as polyphenols as described before [29]. In this assay adapted to a micro plate, 100 μL of açaí extract was added to ABTS+• solution and the absorption was measured spectrophotometrically at 734 nm at 30 °C 10 min after initial mixing and is presented as the Trolox equivalent used as standard reference.

The other radical scavenging assay involves the free radical 2,2-diphenyl- 1-picrylhydrazyl or DPPH•, which reacts with an antioxidant molecule resulting in a discoloration of the solution, measured at 515 nm [30], and adapted to a micro-plate UV-Vis reader. The radical 150 μM DPPH• solution was prepared by mixing the DPPH reagent (Sigma-Aldrich, St. Louis, MO, USA) in 80% methanol (HPLC grade, Sigma-Aldrich, St. Louis, MO, USA). In a 96 well-plate, 180 μL of the radical solution was added to 20 μL of the açaí extract, standards or 70% methanol, which was used as a blank. The plate was stored in the dark for 40 min and read at 515 nm. The results were also quantified using a calibration curve of Trolox (Sigma-Aldrich, St. Louis, MO, USA) over the range of 100 to 500 μM.

### 2.4. Elemental Analysis

The determination of the total elemental composition of the açaí samples was performed using an inductively coupled plasma mass spectrometry (ICP-MS). Samples of 0.25 g were fully digested at 500 °C for 12 h using a Carbolite AAF 1100 muffle furnace. The resultant ash was homogenised with 1 mL nitric acid (PrimarPlus- Trace Analysis Grade 68%, Fisher Scientific, Loughborough, UK). The digest was diluted with double-distilled deionised water until 25 g. Each final solution was filtered using a 0.45 μm Millex-HA membrane filter (Merck Millipore, Darmstadt, Germany) and the resultant solutions were analysed. The instrumental performance was validated in this study by means of the comparative analysis of two water certified reference materials (CRMs), namely, NIST SRM 1640a (National Institute of Standards and Technology, Gaithersburg, MD, USA) and the TMDA 54.4 (National Water Research Institute, Burlington, ON, Canada). The results were quantified using a calibration curve of Ca, Mg, Mn, Fe, Zn and Cu standards (1000 mg L/1 of stock solution, BDH^®^, Aristar^®^, Lutterworth, UK) over the range of 1 to 1000 μg/L.

### 2.5. Biological Assays on RAW 264.7 Macrophage Cells

#### 2.5.1. Macrophage Cell Culture

The mouse macrophage cell line RAW 264.7 (ATCC TIB-71, American Type Culture Collection; Manassas, VA, USA) used in this study was maintained in Dulbecco’s modified Eagle’s medium (DMEM, Life Technologies, New York, NY, USA), supplemented with 100 IU/mL penicillin/100 μg/mL streptomycin (Fisher Scientific, Pittsburg, PA, USA) and 10% fetal bovine serum (Life Technologies, New York, NY, USA) at a density not exceeding 5 × 10^5^ cells/mL and was maintained at 37 °C in a humidified incubator with 5% of carbon dioxide prior to the analysis.

The cell line Human Dermal Fibroblast cells (adult)–HDFa (Invitrogen C-013-5C, Thermo Fisher Scientific Massachusetts, Waltham, MA, USA) was maintained in Medium 106 (Invitrogen M-106-500, Thermo Fisher Scientific Massachusetts, Waltham, MA, USA) supplemented with Low Serum Growth Supplement–LSGS (Invitrogen S-003-10, Thermo Fisher Scientific Massachusetts, Waltham, MA, USA) and 1% of the antibiotic penicillin /streptomycin solution; 10,000 IU/10,000 μg/mL (Fisher MT-30-002-CI, Fisher Scientific, Pittsburgh, PA, USA) at a density minimum of 2.5 × 10^4^ viable cells/mL and was maintained at 37 °C in a humidified incubator with 5% of carbon dioxide prior to the analysis.

#### 2.5.2. Cell Viability Assay

The RAW 264.7 cells were seeded in a 96-well plate for the viability assay. Cell viability was measured by the MTT (3-(4,5-dimethylthiazol-2-yl)-2,5-diphenyl-tetrazolium bromide) assay as previously described [31]. In short, the cells were seeded in a 96-well plate and treated with two different doses of the açaí extracts, 50 and 250 μg/mL. After incubation, the media were discarded and 100 μL of DMSO (dimethyl sulfoxide, Sigma-Aldrich, St. Louis, MO, USA) was added to dissolve the purple crystals and quantified spectrophotometrically at 550 nm using a microplate reader SynergyH1 (BioTek, Winooski, VT, USA).

#### 2.5.3. Nitric Oxide Radical Inhibition Assay

The ability of açaí extractions to inhibit nitric oxide (NO) radical formation was determined [26]. The nitrite production, a stable end product of NO production in activated macrophages, was determined colorimetrically at 540 nm and read on the microplate reader. A total of 100 μL of cell culture media was added to 100 μL of Griess reagent (1% sulfanilamide and 0.1% naphthylethylenediamine in 5% phosphoric acid, Promega, Fitchburg, WI, USA), and the mixture was incubated at room temperature for 10 min. The cells were treated with 50 μg/mL of the açaí extracts. The absorbance was compared against a set of sodium nitrite standards (Promega, Fitchburg, WI, USA).

#### 2.5.4. Reactive Oxygen Species Assay

In order to determine the in vitro reactive oxygen species (ROS) generation, a fluorescent dye protocol was adapted [32]. The RAW 264.7 macrophage cells were seeded in a 24-well plate and incubated overnight at 37 °C. Cells were then charged with a 50 μM solution of dichlorodihydrofluorescein diacetate acetylester (H2DCFDA, Molecular Probes, Eugene, OR, USA), freshly prepared in sterile phosphate-buffered saline (PBS, Sigma-Aldrich, St. Louis, MO, USA) for 30 min. The fluorescent medium was aspirated, and cells were exposed to extracts (final concentration at 50 μg/mL) and lipopolysaccharide (LPS, from *Escherichia coli* 026:B6, Sigma-Aldrich, St. Louis, MO, USA, 10 ng/mL), incubated for 24 h and the fluorescence of 2′,7′-dichlorofluorescein (DCF) was measured at 485 nm (excitation) and 515 nm (emission) on the microplate reader. As a positive control, the dexamethasone (DEX, Sigma-Aldrich, St. Louis, MO, USA) was used at 10 μM.

#### 2.5.5. Cell Migration Assay

The cell migration assay was performed using the OrisTM Cell 2-D migration of adherent cells assay kit (AMSBio, Cambridge, MA, USA), where plastic stoppers were used to simulate a wound [11]. Furthermore, the cells were dyed with NucBlue^®^ Live Cell Stain (Thermo Fisher Scientific Massachusetts, Waltham, MA, USA), which is a reagent that binds to DNA and can be excited by UV light at 360 nm, with an emission maximum at 460 nm. The stoppers were applied to a 96-well plate, where suspended dyed cells and the açaí extracts (50 μg/mL) were added to each well, except for the controls that were run at the same time. For the blank readings, only the medium was added to the wells; 10% of fetal bovine serum (FBS, Thermo Fisher Scientific Massachusetts, Waltham, MA, USA) was used as a positive control; and, for the full cell readings, the stoppers were never inserted to the wells, allowing the cells to migrate. The plate was incubated for 2 h and the fluorescence was read, and blank corrected, on the micro-plate reader SynergyH1 (BioTek, Winooski, VT, USA) at time zero. Moreover, the images of the cells were captured using the microscope EVOS FL Cell Imaging System (Thermo Fisher Scientific Massachusetts, Waltham, MA, USA). The cells were further incubated for another 48 h and the migration of the cells were evaluated both via florescence and imaging.

### 2.6. Statistical Analysis

Statistical analyses were performed by using the GraphPad Prism 4.0 (GraphPad Software Inc., La Jolla, CA, USA). The data were analysed by one-way analysis of variance (ANOVA) followed by Turkey’s multiple comparison tests when comparing the differences between experimental groups and Dunnett’s multiple comparison tests when comparing the difference between the control and test groups. All the data were presented as mean ± SEM and the significant difference was accepted if *p*-value was less than 0.05.

## 3. Results and Discussion

### 3.1. Chemical Analysis and Antioxidant Activity

The chemical composition and antioxidant activity of açaí samples are presented in Table 1. From these data, it is clear that the samples that were de-fatted (i.e., removal of the oil fraction) show a significantly higher total polyphenol content for the non-commercial samples (*p* < 0.0001, *n* = 16, α = 0.05). The removal of the oil fraction results in the pre-concentration of the total polyphenol content in the de-fatted samples. Moreover, the oil does not have a significant level of total polyphenols, as shown and confirmed by previous studies [2].

**Figure 1:**
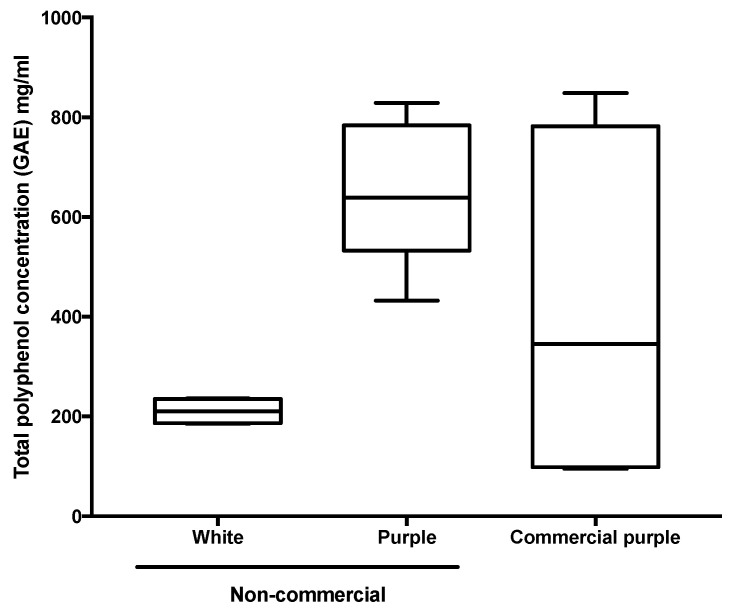
Box plots of the total polyphenol content (gallic acid equivalent mg/g) of the açaí extracts determined using the Folin–Ciocalteu assay. The values relate to the type of sample (non-commercial purple, *n* = 6; non-commercial white and commercial *n* = 4; *n* is the number of samples).

Figure 1 presents a comparison of the total phenolic content of non-commercial and commercial purple and white açaí freeze-dried berries. The purple non-commercial samples have a significantly higher amount of total polyphenol content in comparison with the white samples (*p* = 0.0003, *n* = 9, α = 0.05). Furthermore, the commercial purple berries have a higher level of variability (standard error of the mean of 9.26 GAE mg/g) when compared with the purple pure açaí berries (2.90 GAE mg/g). The total polyphenol content of the açaí pulp observed in this study is in accordance with the range reported in the literature for açaí purple berries [11,33,34], white berries [5] and oil [2]. The non-commercial purple and commercial samples from São Paulo represent an average level of the total polyphenol content, in comparison with other 18 non-traditional Brazilian fruits, based on reported classification [35]. Açaí presented four times higher extractable polyphenol levels than fruits such as *caju* and *caja*—similar to *jaboticaba*, but half of the levels presented by *camu-camu* and *acerola* [34].

The main advantage of consuming açaí rather than the other fruits is the easy availability of açaí in all regions of the country (as a frozen pulp). As such, the consumption of the processed frozen pulp is not dependent on seasonality [36], in contrast to other Brazilian fruits and seasonal berries. In comparison with other traditional berries available on the international market, the non-commercial purple açaí and the commercial powder SP had similar total polyphenol content as cranberries, higher than raspberries and grapes and lower than blackberries, bilberries and blackcurrants [37]. The non-commercial white and the other commercial açaí samples presented significantly lower values than those reported for other typical berries [37].

The total flavonoid content of açaí pulp extractions has a similar pattern to that for the total polyphenol content, as it would be expected because the flavonoids are a subclass of polyphenols. The data are also in agreement with the available literature for commercial açaí samples [35,38].

**Figure 2:**
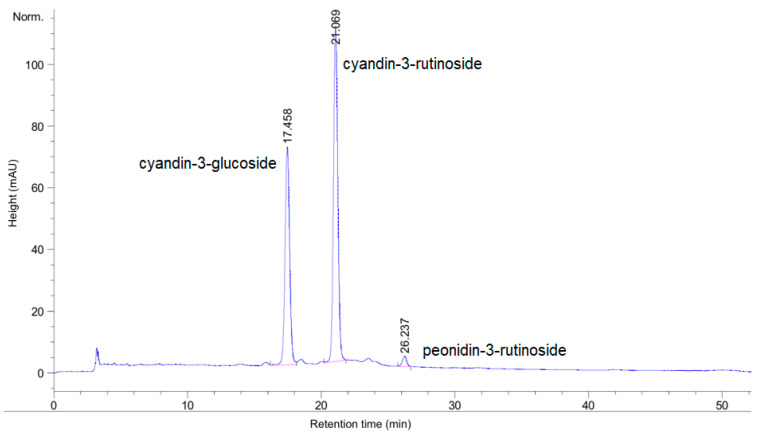
Determination of the anthocyanin content of a purple non-commercial açaí sample following methanolic extraction and using an high-performance liquid chromatography instrument with a photodiode array detector (HPLC-DAD) chromatograph (at 520 nm). The anthocyanin peaks are cyanidin-3-glucoside (retention time, t_R_ = 17.458 min), cyanidin-3-rutinoside (t_R_ = 21.069 min) and peonidin-3-rutinoside (t_R_ = 26.237 min). Norm.: Figure scale is normalized to the highest peak.

Figure 2 shows the HPLC-DAD chromatogram at 520 nm used to determine the total anthocyanin content of the açaí samples. The anthocyanin profile presented in Figure 2 of a purple non-commercial açaí sample was the same for all purple açaí samples, and no peaks were detected for the white samples. The anthocyanin peaks found in the purple (non-commercial and commercial) samples are cyanidin-3-glucoside (retention time, t_R_ = 17.458 min), cyanidin-3-rutinoside (t_R_ = 21.069 min) and peonidin-3-rutinoside (t_R_ = 26.237 min), respectively. The identification of the anthocyanin peaks was performed using a cyanidin 3-glucoside reference standard and the peak retention order by the method previously proposed [39]. The anthocyanin profile and content of the purple açaí berries are in agreement with previous studies [34,40]. The purple açaí samples were the only extracts that showed an anthocyanin profile, as may be expected from the purple colour of the material, which is due to the presence of anthocyanin molecules [41]. In contrast, the white açaí berries did not show any significant levels of anthocyanins. Secondly, the pure açaí powder, commercially purchased in the UK, did not show any significant anthocyanin peaks and was below the limit of detection. This implies that the UK sample might have had some other material added to it, that the chemicals might have been lost during processing, or that the samples come from different locations or climates influencing the concentration of the phytochemicals of the berries [11,42]. In comparison with other typical berries, the total anthocyanin content for the non-commercial purple açaí samples ranges from 10.20 to 14.33 mg/g and for the commercial purple açaí samples 3.59 to 4.70 mg/g. Therefore, açaí would occupy the fourth position in ranking of the berries in terms of the total anthocyanin content of the fruit, behind blackcurrant, blueberry and blackberry [37].

The total proanthocyanidins (PAC) of the açaí samples were evaluated following the DMAC assay. PACs are oligomeric flavonoids [43] and previous studies have shown that the polymers (more than 10 units) are the major PACs in freeze-dried açaí [9]. The higher anthocyanin content and lower PAC was found in the purple açaí samples compared to our previous non-processed samples [11]. It might be due to the harvested season and locations. The difference in the PAC levels between the purple and white samples was lower than that for the other assays presented in this study. This supports the claim that PAC could be the class of polyphenols responsible for the antioxidant activity of the white açaí berries [7]. The reported values of PAC levels for açaí obtained in other studies are in agreement with the data in this study for purple açaí samples [44]. In comparison with other typical super-fruits, the values in açaí are similar to the values for blueberry and cranberry but are slightly higher than that for pomegranate and significantly lower than cocoa seeds [44].

The antioxidant activity results (ABTS and DPPH assay) were presented in Table 1. The data show a significantly higher level of antioxidants for most of the samples, with the exception of the oil extraction samples. The white and the commercial açaí samples purchased in the UK have significantly lower levels of antioxidant activity when compared to the non-commercial purple açaí berries (one-way ANOVA, *p* < 0.0001, *n* = 40, α = 0.05). In addition, the commercial açaí bought in the UK have a significantly lower activity when compared to the other commercial purple samples (one-way ANOVA, *p* < 0.0001, *n* = 23, α = 0.05). The data relating to the antioxidant activity of the açaí extracts are in agreement with the range of results reported in the literature for açaí samples [33,34,45]. The non-anthocyanin phenolic profile and levels of white açaí were found to be similar to the purple berries [5]. Samples of white açaí have antioxidant properties, such as scavenging capacity and inhibition of nitroso compounds formation [5]. The presence of non-anthocyanins phenolics and PAC compounds could be responsible for the antioxidant activity presented in this research. Furthermore, in comparison with other 18 non-traditional Brazilian fruits, açaí presented an average antioxidant activity [35].

The purple açaí samples have a significantly higher antioxidant activity (ABTS and DPPH) when compared to the white açaí, explained by the higher levels of PAC, total anthocyanins, and phenolics, including flavonoids. The antioxidant activity of the white açaí could be related to the PAC levels present in the samples. The data from the commercial samples would suggest that the industrial processing of açaí has an impact on the antioxidant compounds of the berry. In general, açaí is a good source of anthocyanins, PAC and phenolic compounds when compared to other typical Brazilian fruits and is available as a frozen pulp all year.

### 3.2. Elemental Analysis

The total elemental composition of the açaí samples was determined by ICP-MS. The calcium, magnesium, manganese, iron, zinc and copper—essential minor and trace elements found in significant levels when compared to other fruits are presented in Table S1 (Supplementary Material). The difference between the elemental concentrations of the non-commercial whole and de-fatted samples follow the same trend as the previous data reported in the previously sections. Overall, for both white and purple açaí pulp, all of the elemental values for the de-fatted samples are slightly higher than the whole material due to the removal of the oil fraction. The enhancement of the elemental levels in the defatted samples is on average 28% for purple and 38% for white samples. Interestingly, the commercial pulp and powder products purchased in São Paulo have similar elemental levels. However, the powered sample purchased in the UK has either been subjected to a different process or has been adulterated as the elemental levels are typically 30% of the Brazilian powdered samples, as presented in the previous analysis of this study. The commercial samples, especially the açaí pulp (purchased in São Paulo), have lower (and a larger spread) elemental levels than the non-commercial samples. This may be due to the origin of the berries used in the commercial sample or the processing method used (which are both unknown).

**Figure 3:**
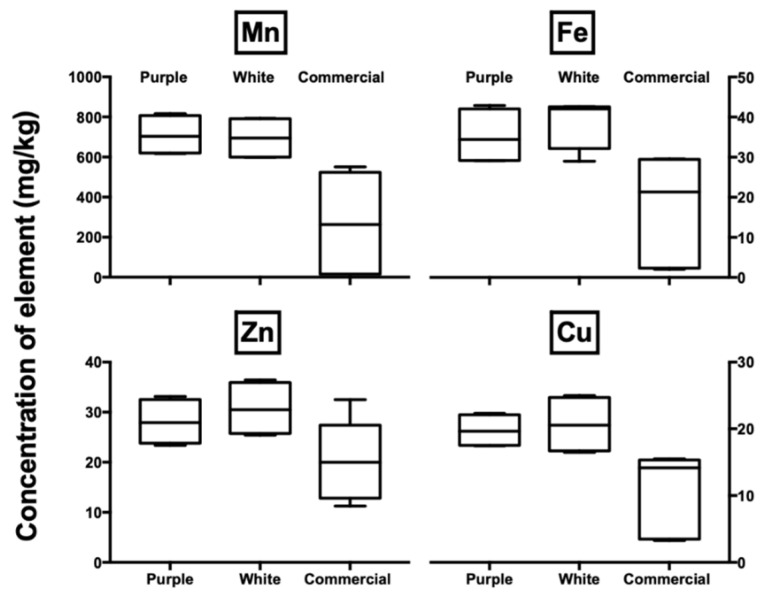
Box plots of the total elemental content of trace elements (mg/kg dry weight) of açaí pulp samples using inductively coupled plasma mass spectrometry (ICP-MS) relating to the type of sample (non-commercial: purple *n* = 6; and white *n* = 4; and commercial: purple *n* = 4; *n* is the number of samples). The commercial sample is a combination of pulp SP and powders SP and UK.

Figure 3 graphically reports the data (as box plots) for the non-commercial purple or white and commercial purple açaí samples. In the box plots, the pulp SP, powder SP and powder UK samples were combined as commercial samples, causing a spread of the target elemental concentrations. The levels of manganese are at least three times higher than those reported in other studies [17,46,47]. Iron, copper and zinc have similar levels to those reported by a previous study that analysed a commercial sample of açaí pulp purchased in Belém (Amazon-Pará) [17]. When compared to the other 47 typical fruits, açaí has the highest content of manganese, the 3rd highest of calcium, 4th of copper, 6th of magnesium, 11th of zinc and 13th of iron content [46]. In comparison to other fruits, açaí represents good levels of manganese and high levels for the other elements (Ca, Mg, Fe, Zn and Cu), with the exception of *graviola* and *pequi* [46]. The typical daily fruits of a Brazilian diet include mango and papaya, which, when compared with the results for açaí, have lower levels of the essential elements [46]. The purple and white açaí have similar elemental levels. There was no significant difference between the elemental levels of non-commercial purple and white açaí (purple and white whole: *t*-test, *p* = 0.8013, *n* = 15, α = 0.05; purple and white de-fatted: *t*-test, *p* = 0.1622, *n* = 15, α = 0.05). This suggests that the samples might come from the same region, or from a similar soil composition [48].

### 3.3. Biological Assays

#### 3.3.1. Cell Viability Assay with MTT

The potential toxicity of the açaí extracts was determined using the cell viability assay, as described. 2.5% dimethyl sulfoxide (DMSO) was used as a positive control, due to its known toxicity to the cells at higher concentrations, where the viability of the cells decreases by half when treated with 5 μL of DMSO. Neither of the açaí samples showed a significant toxicity response, even at the high concentration of 250 μg/mL (One-way ANOVA, *p* = 0.0757, *n* = 31, α = 0.05).

#### 3.3.2. Nitric Oxide (NO) Inhibition

In order to evaluate the cellular antioxidant activities of the açaí extracts, the amount of nitric oxide produced and released to the media of the cells was measured. The cells were induced by lipopolysaccharides (LPS) of bacteria to respond to an inflammatory process [49]. As a positive control, the cells were treated with dexamethasone (DEX), a well-known compound that has an anti-inflammatory activity and inhibits the production of NO. Comparison with the LPS induction study confirmed that neither of the açaí samples (purple or white, and non-commercialised or commercialised) showed any inhibition of the NO production by the extracts at 50 μg/mL (One-way ANOVA, *p* = 0.2705, *n* = 3, α = 0.05). Therefore, the açaí extracts evaluated in this research do not have an effect on the inducible nitric oxide synthase (iNOS). This result contradicts a previous study [50], although different açaí samples (origin, processing method) were used in those studies, potentially leading to different results. Finally, our previous study also showed no inhibition of the NO production by the extracts from industrially processed açaí products but reported significant NO inhibition by the non-processed extracts at a 2-fold higher concentration 100 μg/mL [11]. The negative correlation between the PAC levels and NO production has been previously described [11,22]. The PAC levels of the açaí samples presented in this study are similar to the levels reported for the industrially processed açaí products [11], suggesting that the PAC in these samples have been degraded.

#### 3.3.3. Radical Oxygen Species (ROS)

**Figure 4:**
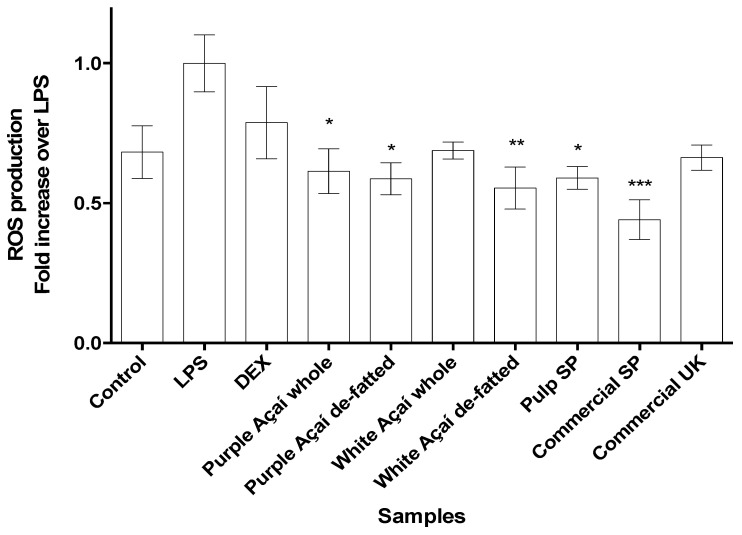
Radical oxygen species (ROS) production in RAW 264.7 macrophage cells stimulated with lipopolysaccharide (LPS). The cells were treated with 50 μg/mL açaí extracts and dexamethasone (DEX). Results are expressed as the mean ± SD, *n* = 3, number of instrumental replicates, * *p* < 0.05, ** *p* < 0.01, *** *p* < 0.001 vs. the LPS-treated group. One-way analysis of variance (ANOVA), Dunnett’s post hoc test.

The in vitro radical oxygen species or ROS assay was used to evaluate the capacity of the açaí extracts to decrease the production of ROS in stressed cells. To this end, the cells were tagged with a fluorescence dye and then induced with LPS and treated with the açaí extracts. The results, as shown in Figure 4, were normalized by the fluorescence levels of the cells induced by LPS only and compared with the treated cells. It is clear that the açaí samples had a positive effect on the inhibition of the ROS generation, confirming the antioxidant activity shown in the chemical assays. The purple and white açaí samples presented in the Figure 4 are a combination of the non-commercial whole and de-fatted samples, as they did not result in any significant difference using the t-test (for purple *p* = 0.9903, *n* = 4, α = 0.05 and white, *p* = 0.4669, *n* = 4, α = 0.05). When compared with the LPS-induced cells, all the açaí samples showed a statistically significant difference (one-way ANOVA, r = 0.55, *p* = 0.0012, *n* = 30, α = 0.05) confirming the antioxidant activity shown in the chemical assays [1]. Berries with high phenolic levels have previously demonstrated the ability to decrease ROS production in cells [51,52]. In general, polyphenols can act as antioxidants modulating the damage in cells with increased ROS levels [53]. These results confirm that the açaí berry is effective in controlling oxidation reactions, acting as an antioxidant agent in cells.

#### 3.3.4. Cell Migration Assay

**Figure 5:**
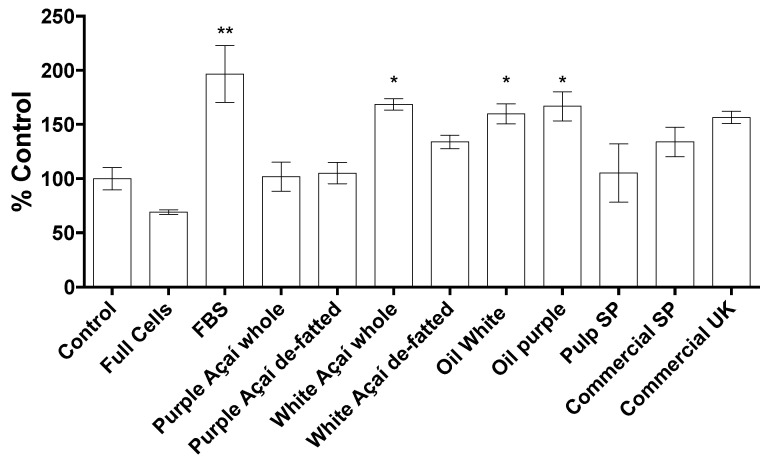
Effect of açaí extract on the migration of human dermal fibroblast cells (adult). The cells were treated with 50 μg/mL açaí extracts or 10% fetal bovine serum (FBS). Results are expressed as the mean ± SD, *n* = 3; *n*, number of instrumental replicates, * *p* < 0.05, ** *p* < 0.01 vs. the control group. One-way ANOVA, Dunnett’s post hoc test.

The results for the wound healing experiment are shown in Figure 5 as the difference between the fluorescence values of *t* = 48 h minus *t*= 0 h of incubation. The data were normalised to the values of the positive control, 10% FBS. Furthermore, the purple açaí results combine the non-commercial purple samples whole and de-fatted (no significant difference, *p* = 0.8449, *n* = 8, α = 0.05); finally, the commercial pulp SP, commercial SP and commercial UK samples did not show a significant difference using a one-way ANOVA test (*p* = 0.1148, *n* = 21, α = 0.05).

**Figure 6:**
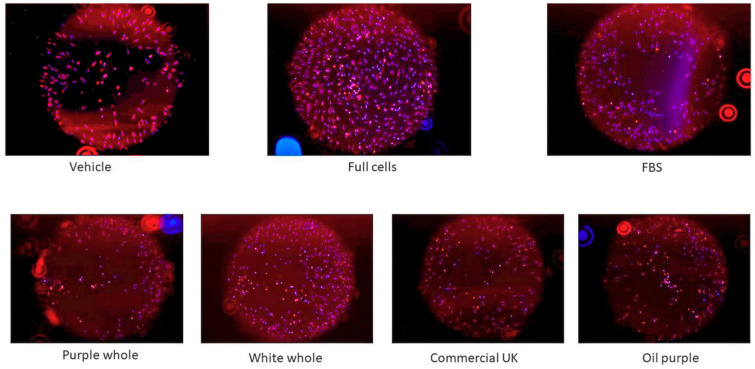
Progress of human dermal fibroblast cells (adult) after 48 h of treatment with 50 μg/mL açaí extracts or 10% fetal bovine serum (FBS). The fluorescent images were captured by EVOS^®^ FL Auto Cell Imaging System. The vehicle used was 80% methanol; full cells correspond to wells seeded without stopper and FBS was used as a positive control.

Figure 6 shows a visual difference of the migration of the cells between time 0 and after 48 h of incubation, following the treatment with the açaí samples. The non-commercial white açaí whole sample was the sample that showed the highest potential of wound healing, as shown in Figure 4 (about 1.75-fold increase over control).

The results for this assay confirm the potential of the açaí extracts as a wound healing agent. In contrast to the other experiments reported above in this study, the samples where the oil was present exhibited a positive effect on the wound healing. This indicates that the açaí oil plays an important role in the wound healing process. Previous studies that analysed the composition of the fatty acids of the purple and white açaí oil, reported levels of 60% oleic acid, 22% of palmitic acid, 12% of linoleic acid, 6% of palmitoleic acid and traces of other fatty acids [2]. Purple and white açaí berries also contain flavonoids and other phenolic compounds, such as vanylic acid, that have antioxidant properties [54]. Plant oils can easily penetrate the lipid structures of the skin and influence the cell membrane proteins in order to promote conformational modifications [55]. It has already been established that fatty acids play an important function in the migration of cells, modulating skin proliferation and immune responses [21]. Iron and zinc are positive factors that also help to improve or accelerate the wound healing process [21]. The wound closure can also be effected by the regulation of skin inflammation and permeability [55], confirming the açaí potential as a wound healing agent not only due to the oil content but also the anti-inflammatory benefits and high levels of iron and zinc. Direct effects of palmitoleic acid on wound healing was confirmed in the animal models and clinical studies [56,57]. Finally, açaí oil in nanoemulsions has been applied as a potential treatment for melanoma skin cancer [58].

## 4. Conclusions

In summary, the purple non-commercial açaí samples showed higher concentrations of total polyphenol, flavonoid, anthocyanin and proanthocyanidin in comparison with the white and commercial samples. Moreover, the commercial purple açaí samples had a higher variability when compared with the non-commercial purple berries. The removal of the oil fraction showed a concentration effect in the analysed compounds. Cyanidin-3-glucoside and cyanidin-3-rutinoside were the major anthocyanins in the purple samples and not present in the white and one commercial samples. The non-commercial purple açaí had the highest levels for all antioxidant analyses when compared to the other samples. The calcium, magnesium, manganese, iron, zinc and copper—essential minor and trace elements—were found in significant levels in açaí samples in comparison with other fruits. The strong antioxidant effect of the açaí berries was confirmed in cells by the inhibition of the radical oxygen species production, although the samples did not show any inhibition of the nitric oxide production. Furthermore, the addition of açaí extracts and oils enhanced the levels of migration activity in human fibroblast cells. This research shows that açaí is an important source of bioactive compounds and has a potential as a wound healing and antioxidant agent.

## Figures and Tables

**Table 1:** Polyphenol, flavonoids, anthocyanins and proanthocyanidins content and antioxidant capacities of açaí.

	Samples	Total Phenolics ^1^	Total Flavonoids ^2^	Total Anthocyanins ^3^	Total Proanthocyanidins ^4^	ABTS ^5^	DPPH ^6^	
**Non-commercial**	Purple açaí whole	32.00 ± 1.03 ^c^	6.39 ± 1.23 ^b^	10.20 ± 0.24 ^b^	6.10 ± 2.09 ^a^	438.0 ± 17.5 ^b^	336.0 ± 72.0 ^ab^	
	Purple açaí de-fatted	39.40 ± 1.67 ^b^	8.05 ± 0.81 ^a^	14.33 ± 0.58 ^a^	5.06 ± 0.68 ^ab^	529.0 ± 57.0 ^a^	419.0 ± 69.5 ^a^	
	White açaí whole	9.40 ± 0.70 ^f^	2.12 ± 0.27 ^d^	<0.01	3.96 ± 2.39 ^ab^	83.0 ± 9.7 ^de^	53.2 ± 15.1 ^d^	
	White açaí de-fatted	11.70 ± 0.24 ^e^	2.38 ± 0.35 ^d^	<0.01	2.60 ± 0.49 ^ab^	101.0 ± 16.2 ^de^	67.4 ± 10.7 ^d^	
	Oil white	2.74 ± 1.13 ^g^	1.42 ± 0.96 ^de^	<0.01	1.54 ± 0.22 ^ab^	<15.3	<7.4	
	Oil purple	1.68 ± 0.52 ^g^	0.87 ± 0.38 ^e^	<0.01	3.40 ± 1.42 ^ab^	<15.3	<7.4	
**Commercial**	Pulp SP	28.30 ± 0.64 ^e^	5.00 ± 0.68 ^c^	3.59 ± 0.12 ^c^	4.75 ± 1.58 ^ab^	310.0 ± 41.4 ^c^	222.0 ± 38.6 ^c^	
	Powder SP	42.40 ± 1.53 ^a^	6.07 ± 1.45 ^bc^	4.70 ± 0.01 ^c^	4.47 ± 0.54 ^ab^	316.0 ± 39.1 ^c^	304.0 ± 43.2 ^bc^	
	Powder UK	5.09 ± 0.42 ^d^	1.88 ± 0.73 ^de^	<0.01	5.48 ± 2.20 ^ab^	55.2 ± 31.4 ^e^	<7.4	

^1^ Total phenolics quantified by Folin–Ciocalteu assay as mg gallic acid equivalent/g; ^2^ total flavonoids quantified by AlCl3 assay as mg quercetin equivalent/g; ^3^ total anthocyanins measured by High-Pressure Liquid Chromatography (HPLC) quantified as mg cyanidin-3-glucoside equivalent/g, ^4^ total proanthocyanidins quantified by 4-dimethylaminocinnamaldehyde (DMAC) assay and HPLC-UV quantification (mg/g); as mg procyanidin B1 equivalent/g; ^5^ radical scavenging activity by 2,2′-azino-bis(3-ethylbenzothiazoline-6-sulphonic acid) (ABTS) as μmol Trolox equivalent/g; ^6^ antioxidant capacity by 2,2-diphenyl- 1-picrylhydrazyl (DPPH) assay as μmol Trolox equivalent/g. Results were expressed as mean ± SD (*n* = 3). All concentrations were calculated based on dry weight. Means with different superscript letters within the same column are significantly different (*p* < 0.05). Sample identification: Pulp SP—frozen açaí pulp from São Paulo; Powder SP—pure açaí powder from São Paulo; Powder UK—pure açaí powder from United Kingdom.

## Supplementary Materials

The following are available online at https://www.mdpi.com/2304-8158/9/10/1481/s1, Table S1: Total elemental levels (mean ± standard deviation) of essential trace elements (mg/kg fresh weight) of açaí pulp samples determined using inductively coupled plasma mass spectrometry (ICP-MS): data relates to the type of sample (non-commercial: purple *n* = 6; and white *n* = 4; and commercial: purple *n* = 4; *n* is the number of samples).
