# Supplementary material for: Chemical Composition and Bioactive Properties of Commercial and Non-Commercial Purple and White Açaí Berries
Source: Foods. 2020 Oct 16;9(10):1481. doi: 10.3390/foods9101481 (PMC7602988; doi:10.3390/foods9101481)
Supplement: Supplementary file 1 [file foods-09-01481-s001.pdf]

foods

MDPI

Table S1. Total elemental levels (mean ± standard deviation) of essential trace elements (mg/kg fresh weight) of açaí pulp samples determined using inductively coupled plasma mass spectrometry (ICP-MS): data relates to the type of sample (non-commercial: purple $n = 6$; and white $n = 4$; and commercial: purple $n = 4$; n is the number of samples).

	Samples						
	Non-Commercial				Commercial		
	Purple Açaí Whole	Purple Açaí De-fatted	White Açaí Whole	White Açaí De-fatted	Pulp SP	Powder SP	Powder UK
Ca	468.82 ± 14.15	527.34 ± 58.07	416.23 ± 2.20	525.11 ± 13.28	165.04 ± 16.12	202.93 ± 17.25	66.17 ± 15.65
Mg	233.23 ± 2.16	272.36 ± 4.15	246.92 ± 12.13	301.77 ± 9.00	202.21 ± 2.75	203.69 ± 2.14	85.92 ± 1.13
Mn	64.06 ± 0.93	80.92 ± 0.77	61.14 ± 0.60	80.89 ± 1.57	26.76 ± 1.51	54.70 ± 1.78	1.62 ± 0.01
Fe	3.01 ± 0.00	4.17 ± 0.12	3.65 ± 0.75	4.30 ± 0.10	3.00 ± 0.04	2.19 ± 0.03	0.23 ± 0.01
Zn	2.49 ± 0.07	3.24 ± 0.17	2.65 ± 0.03	3.62 ± 0.17	2.70 ± 0.65	2.32 ± 0.31	1.27 ± 0.12
Cu	1.81 ± 0.03	2.22 ± 0.01	1.72 ± 0.06	2.50 ± 0.10	1.52 ± 0.05	1.50 ± 0.09	0.35 ± 0.02

Sample identification: Pulp SP—frozen açaí pulp from São Paulo; Powder SP—pure açaí powder from São Paulo; Powder UK—pure açaí powder from United Kingdom.
